# Modeling Growth and Toxin Production of Toxigenic Fungi Signaled in Cheese under Different Temperature and Water Activity Regimes

**DOI:** 10.3390/toxins9010004

**Published:** 2016-12-24

**Authors:** Marco Camardo Leggieri, Simone Decontardi, Terenzio Bertuzzi, Amedeo Pietri, Paola Battilani

**Affiliations:** 1 Department of Sustainable Crop Production—Università Cattolica del Sacro Cuore, Via E. Parmense, 84, 29122 Piacenza, Italy; marco.camardoleggieri@unicatt.it (M.C.L.); simone.decontardi@unicatt.it (S.D.); 2 Institute of Food & Feed Science and Nutrition—Università Cattolica del Sacro Cuore, Via E. Parmense, 84, 29122 Piacenza, Italy; terenzio.bertuzzi@unicatt.it (T.B.); amedeo.pietri@unicatt.it (A.P.)

**Keywords:** *Aspergillus*, *Penicillium*, mycotoxin, ochratoxin, sterigmatocystin, roquefortine

## Abstract

The aim of this study was to investigate in vitro and model the effect of temperature (T) and water activity (*a_w_*) conditions on growth and toxin production by some toxigenic fungi signaled in cheese. *Aspergillus versicolor*, *Penicillium camemberti*, *P. citrinum*, *P. crustosum*, *P. nalgiovense*, *P. nordicum*, *P. roqueforti*, *P. verrucosum* were considered they were grown under different T (0–40 °C) and *a_w_* (0.78–0.99) regimes. The highest relative growth occurred around 25 °C; all the fungi were very susceptible to *a_w_* and 0.99 was optimal for almost all species (except for *A. versicolor*, *a_w_opt* = 0.96). The highest toxin production occurred between 15 and 25 °C and 0.96–0.99 *a_w_*. Therefore, during grana cheese ripening, managed between 15 and 22 °C, ochratoxin A (OTA), penitrem A (PA), roquefortine-C (ROQ-C) and mycophenolic acid (MPA) are apparently at the highest production risk. Bete and logistic function described fungal growth under different T and *a_w_* regimes well, respectively. Bete function described also STC, PA, ROQ-C and OTA production as well as function of T. These models would be very useful as starting point to develop a mechanistic model to predict fungal growth and toxin production during cheese ripening and to help advising the most proper setting of environmental factors to minimize the contamination risk.

## 1. Introduction

Several microorganisms contribute to the features of the final product during cheese-making [1]. The starter microbiota, usually artificially inoculated, is mainly composed of lactic acid bacteria such as *Lactococcus lactis* [2], which starts the cheese-making process by producing lactic acid and allowing the syneresis of the curd. Moreover, the starter culture degrades the proteins and may produce CO_2_ in some processes [3]. The secondary microbiota, mainly coming from the environment, or added as for blue cheese [4], becomes dominant after changes in the substrate, i.e., loss of water from the curd, increased salt and pH [5]. Several types of organisms, like salt-tolerant bacteria, yeasts and filamentous fungi [6,7,8] contribute to create the sensorial and nutritional characteristics of the final product with their proteolytic and lipolytic activities [4,7]. Some fungi, like *Penicillium camemberti* and *P. roqueforti*, are well known as ripening agents in appreciated cheeses [9,10,11]. Unfortunately, other fungi, e.g., *P. nordicum*, can act as spoiling agents and/or mycotoxin producers on products of animal origin [12,13,14], cheese included (Table 1).

Fungal activity is modulated by abiotic and biotic factors [15]; however, knowledge of the ecological needs of fungal mycoflora associated with cheese is poor [16]. Some studies have considered the role of temperature (T) on mycotoxin production by cheese-related molds, but generally only a few temperatures and few mycotoxins were considered [17]. Some data are available, regarding citrinin (CIT; [18]), cyclopiazonic acid (CPA; [19,20]), Penitrem A (PA; [21,22]), PR-Toxin [17,23,24,25] and roquefortine C (ROQ-C; [20,23,24]) under defined T regimes. The role of water activity (*a_w_*) and pH has been poorly studied [16]; ochratoxin A (OTA) production by *P. verrucosum* on YES medium under different pH and *a_w_* regimes, reported by Schmidt-Heydt, et al. [26], is the only research available.

Therefore, the aim of this study was to investigate and model the ecological needs of some fungi frequently reported as cheese contaminants [27] or commonly used as cheese ripening agents, focusing both on growth and mycotoxin production under different T and *a_w_* regimes. *Penicillium camemberti*, *P. citrinum*, *P. crustosum*, *P. nalgiovense*, *P. nordicum*, *P. roqueforti*, *P. verrucosum* and *Aspergillus versicolor* were considered. In a preliminary study, aimed at describing the fungal population associated with cheese during ripening, most of these species were found on the rind of grana type cheeses, sampled during the long aging period.

This is a preliminary study and will contribute to the development of a mechanistic model for the prediction of mycotoxin contamination in ripening cheese.

## 2. Results

### 2.1. Role of Temperature, Water Activity and Incubation Time on Fungal Growth

#### 2.1.1. Temperature

**Figure 1:**
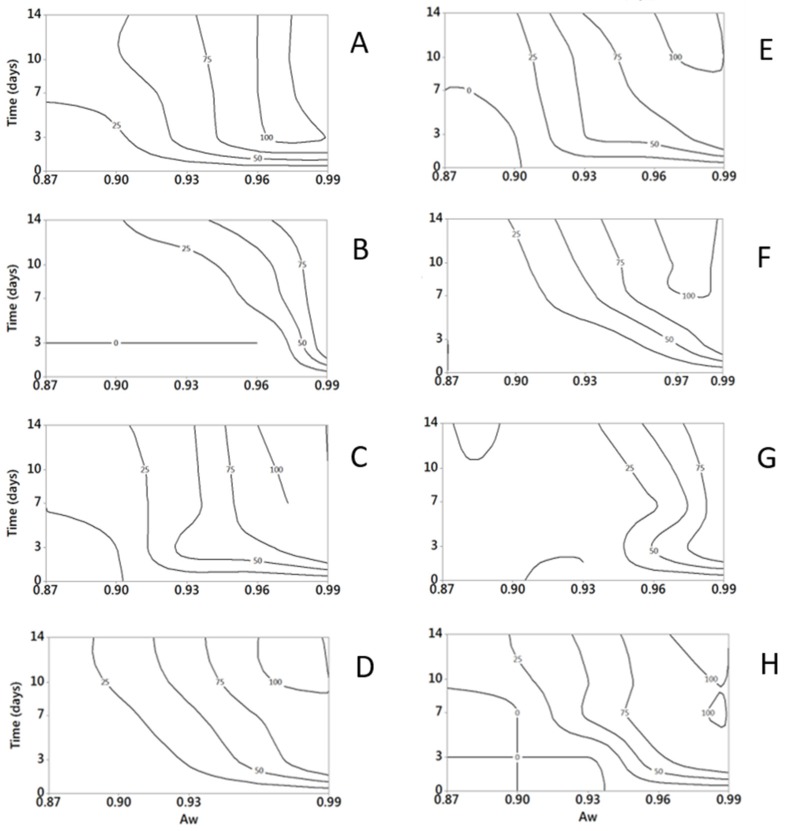
Surface response curves of fungal relative growths (expressed as percentage on the maximum growth, numbers on the isoplethes) at different incubation times (3, 7, 10, 14 days) under different T regimes (0–40 °C, step 5 °C; *a_w_* = 0.99). (**A**) *A. versicolor*; (**B**) *P. camemberti*; (**C**) *P. citrinum*; (**D**) *P. crustosum*; (**E**) *P. nalgiovense*; (**F**) *P. nordicum*; (**G**) *P. roqueforti*; (**H**) *P. verrucosum*.

The effect of T on fungal growth, at different incubation times (0–14 days), is shown in Figure 1 using surface response curves of relative growth; maximum diameter of fungal colony after 1 day incubation is also reported in Table 2.

Fungal growth occurred from 5 to 30 °C for *P. nordicum* and *P. verrucosum*, from 5 to 35 °C for *P. citrinum*, *P. crustosum*, *P. nalgiovense* and *P. roqueforti*, from 10 to 30 °C and from 5 to 25 °C, for *A. versicolor* and *P. camemberti*, respectively. The highest relative growth occurred with T = 25 °C for the majority of fungi, with the exceptions of *P. citrinum* (T = 30 °C), *P. verrucosum* and *P. nordicum* (T = 20 °C). In Table 2, maximum colony growth values after 10-day of incubation are reported for all the selected fungi. This incubation time was considered because one of the fungi (*P. roqueforti*) reached the maximum possible diameter of 5.5 cm.

#### 2.1.2. Water Activity

**Figure 2:**
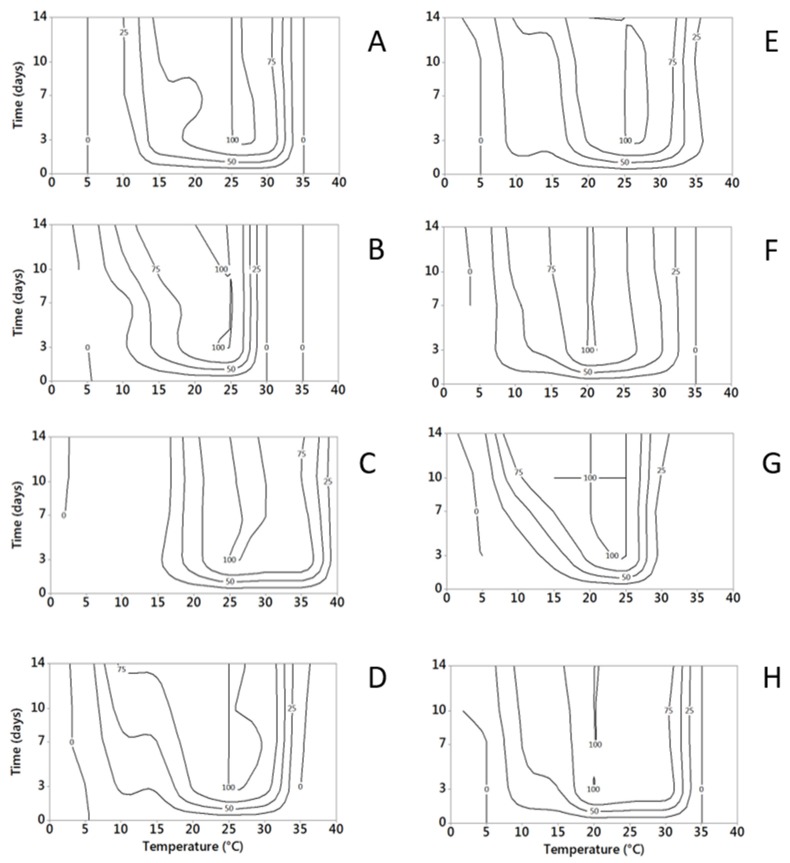
Surface response curves of fungal relative growth (expressed as percentage on the maximum growth, numbers on the isoplethes) at different incubation times (3, 7, 10, 14 days) under different *a_w_* regimes (0.87–0.99; step 0.03; T = 20 °C). (**A**) *A. versicolor*; (**B**) *P. camemberti*; (**C**) *P. citrinum*; (**D**) *P. crustosum*; (**E**) *P. nalgiovense*; (**F**) *P. nordicum*; (**G**) *P. roqueforti*; (**H**) *P. verrucosum*.

Fungal growth increased with the rise of *a_w_*, within the range considered (0.87–0.99 *a_w_*), as a general trend for all the species (Figure 2); *A. versicolor* and *P. roqueforti* grew from 0.87 *a_w_*, while *a_w_* = 0.90 was the minimum for all the others except *P. camemberti* and *P. crustosum*. Maximum growth was observed at 0.99 *a_w_* for all the species except *A. versicolor*, with 0.96 *a_w_* as optimal. At the optimal *a_w_*, all the fungal colonies reached maximum diameter between 7 and 14 days; the maximum colony diameters after 10 days of incubation is reported in Table 2.

Incubation times of up to 56 day, applied for *a_w_* = 0.87 and *a_w_* = 0.90, showed *P. camemberti*, *P. crustosum* and *P. nordicum* growth only at *a_w_* = 0.90, while a relevant growth was observed for all the other species at both *a_w_* considered.

### 2.2. Modeling the Influence of Temperature and Water Activity on Mycelial Growth

**Figure 3:**
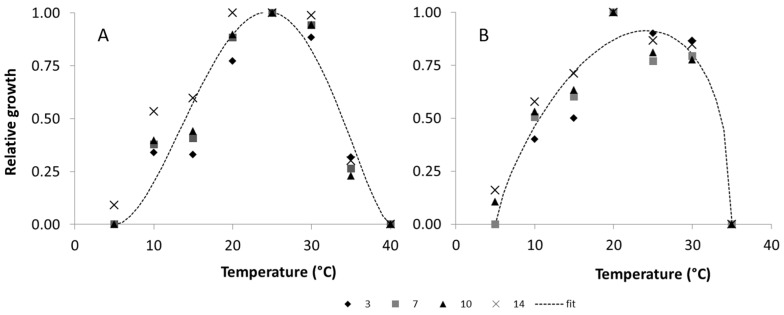
Dynamic of relative growth of (**A**) *P. nalgiovense* and (**B**) *P. verrucosum*, after 3, 7, 10 and 14 days of incubation, at different temperature regimes (0–40 °C). Data were fitted (dotted line) by a Bete function (see Table 3 for equation parameters).

Fungal relative growth for each fungus, in different T regimes, showed a comparable trend at each incubation time (examples in Figure 3). Therefore, 36 mean relative growth values were used for T model fitting (9 T regimes and 4 incubation times, mean of 3 replicates). Regarding *a_w_*, fungal growth decreased under sub-optimal values and 3 day incubation time was not included in data analysis. Instead of 32 mean relative growth values for *a_w_* (8 *a_w_* regimes and 4 incubation time, mean of 3 replicates), were therefore used 15 mean values (5 *a_w_* regimes and 3 incubation time, mean of 3 replicates) for *a_w_* model fitting.

#### 2.2.1. Temperature

**Figure 4:**
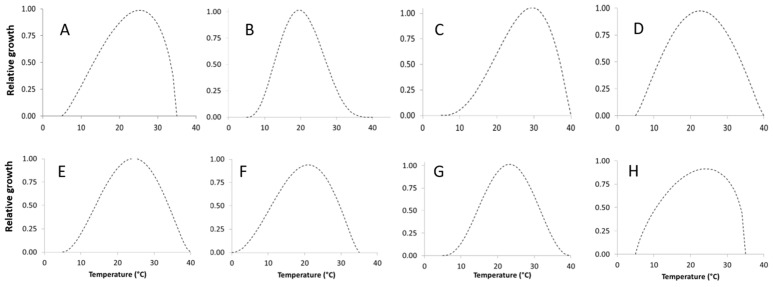
Dynamic of relative growth of the studied fungi, at different temperature regimes (0–40 °C). Data were fitted by a Bete function (see Table 3 for equation parameters). (**A**) *A. versicolor*; (**B**) *P. camemberti*; (**C**) *P. citrinum*; (**D**) *P. crustosum*; (**E**) *P. nalgiovense*; (**F**) *P. nordicum*; (**G**) *P. roqueforti*; (**H**) *P. verrucosum*.

The best fitting of fungal growth data as function of T was obtained, for all fungi considered, by the Bete equation [45] in the form:

(1) $$y={\left(a*{\left(Teq\right)}^{b}*\left(1-Teq\right)\right)}^{c}$$

where *y* is the relative growth of the colonies, *a*, *b* and *c* are the equations parameters and *Teq* is the equivalent T calculated as follows:

(2) $$Teq=\left(T-T_{\min})/(T_{\max}-T_{\min}\right)$$

where *T* is the temperature regime and T_min_ and T_max_ are minimum and maximum temperature, respectively, at which the fungus is able to grow. Parameters, T_min_/T_max_ and R^2^ values are reported in Table 3. The goodness of fit, measured with *R*^2^, was always good, ranging from 0.798 to 0.96. Fungal growth increased from 5 °C (0 °C for *P. nordicum*) to 20 °C (*P. camemberti*, and *P. verrucosum*) or 25 °C (*A. versicolor*, *P. crustosum*, *P. citrinum*, *P. nalgiovense*, *P. roqueforti*), and then quickly decreased (Figure 4). *P. camemberti* and *P. nordicum* showed a very similar behavior, with a fast relative growth up to 25 °C, followed by a rapid decrease (Figure 4).

#### 2.2.2. Water Activity

**Figure 5:**
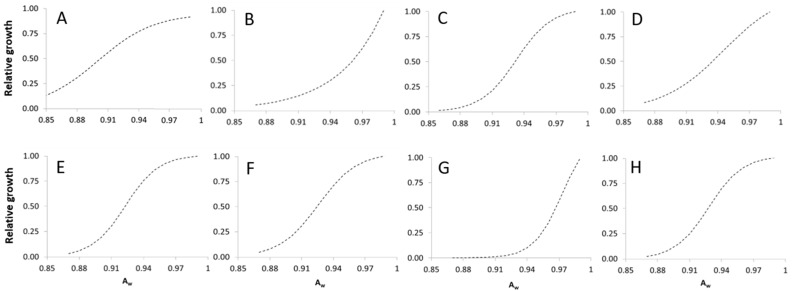
Logistic equations (lines, refer to Table 3 for equation parameters) defining the dynamics of fungal growth at different a_w_ regimes (0.78–0.99). (**A**) *P. versicolor*; (**B**) *P. camemberti*; (**C**) *P. citrinum*; (**D**) *P. crustosum*; (**E**) *P. nalgiovense*; (**F**) *P. nordicum*; (**G**) *P. roqueforti*; (**H**) *P. verrucosum*. (for Figure 5A suitable a_w_ start from 0.78 but the same range of other fungi was used).

The influence of different *a_w_* regimes on fungal growth was well described using a Logistic equation, in the following form:

(3) $$y=c/\left(1+exp^{\left(a\;+\;b*a_{w}\right)}\right)$$

in which *y* is the fungal relative growth, *a*, *b*, and *c* are equation parameters, reported in Table 3. All fungi showed an S-shaped growth, except *P. camemberti* and *P. roqueforti*, with a J-shaped trend [46], without the upper plateau (Figure 5). The functions developed showed a very good fitting to growth data with R^2^ ranging between 0.925 and 0.979.

### 2.3. Modeling the Combined Effect of Temperature and Water Activity on Mycelial Growth

The combined effect of T and *a_w_* was also considered, merging the functions previously developed, as follows:

(4) $$y=a_{T}\;\times Teq^{b_{T}}\times {\left(1-Teq\right)}^{c_{T}}\times c_{a_{w}}/\left(1+exp^{\left(a_{a_{w}}\;+\;b_{a_{w}}*a_{w}\right)}\right)$$

in which *y* is the relative growth, computed referring to the maximum growth observed.

**Figure 6:**
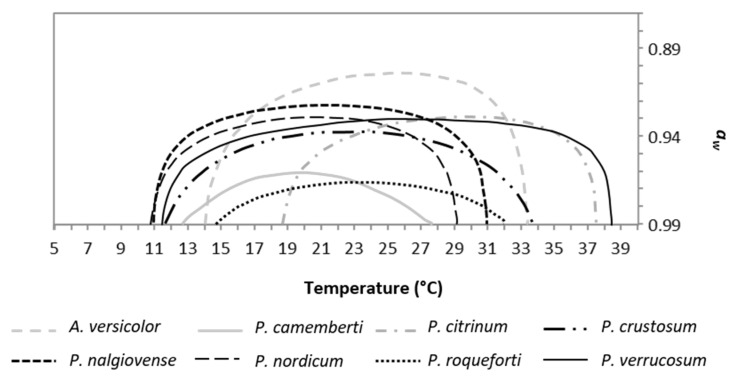
Boundaries, derived from Equation (4), summarizing the combination of T and a_w_ conditions to reach relative growth =0.5 for each fungus considered in the study.

The output was plotted in a single chart (Figure 6), where the curves represent the combination of conditions that allow to reach 50% relative growth, for each fungus, as function of T and *a_w_*.

Focusing attention on T values, the number of fungal species able to grow increased from 10 °C up to 20 °C, then decreased starting from 27 °C. *P. nordicum* started growth from 10 °C, immediately followed by *P. nalgiovense*, *P. verrucosum*, *P. crustosum*, *P. camemberti*, *A. versicolor*, *P. roqueforti* (from 14 °C), and *P. citrinum* (from 19 °C). All fungi were able to grow between 20 and 27 °C. Then, moving to higher temperatures, *P. camemberti* was the first to reduce the relative growth below 50% (T limit around 27 °C), followed by *P. nordicum* (29 °C), *P. nalgiovense* (31 °C), *P. roqueforti* (32 °C), *A. versicolor* and *P. crustosum* (34 °C), *P. citrinum* and *P. verrucosum* (37–39 °C).

Considering *a_w_*, the number of species with growth >50% gradually decreased as *a_w_* values moved from 0.99 to 0.87. *P. roqueforti* showed a relative growth below 50% at 0.97 *a_w_*, followed by *P. camemberti* and *P. crustosum* (0.96 and 0.94 *a_w_* respectively), *P. citrinum*, *P. nalgiovense*, *P. nordicum*, and *P. verrucosum* (around 0.93 *a_w_*) and *A. versicolor* (0.90 *a_w_*).

### 2.4. Influence of Temperature and Water Activity on Mycotoxin Production

All the mycotoxins investigated were detected (µg/L > LOD) and reported in ng/mm^2^ (Table 4), except CPA, PR and CIT in *P. citrinum*, *P. roqueforti* and *P. verrucosum*, respectively. Significant differences in the produced amount of toxins, depending on the fungus and ecological conditions tested, were noticed, except for CIT produced by *P. camemberti*. In particular, the optimum temperature for mycotoxin production was commonly between 20 and 25 °C, and the optimum *a_w_* was 0.99, except for MPA optimally produced by *P. roqueforti* at the combination 20 °C and 0.96 *a_w_*. No mycotoxin was detected in fungal colonies grown with *a_w_* < 0.93 after the 14-day incubation.

### 2.5. Modeling the Effect of Temperature and Water Activity on Toxin Production

#### 2.5.1. Temperature

**Figure 7:**
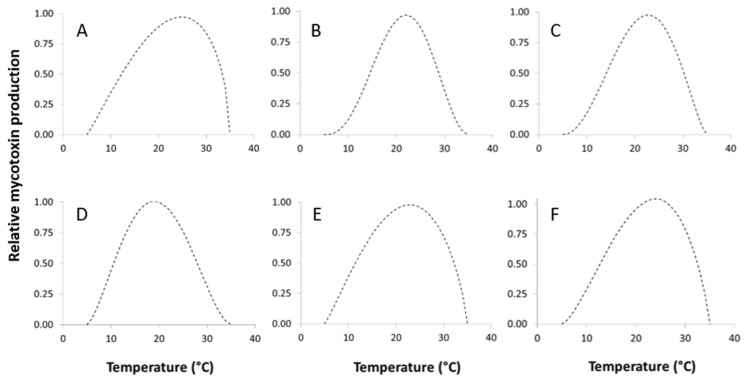
Dynamic of mycotoxins production rate for: (**A**) STC—*A. versicolor*; (**B**) PA—*P. crustosum*; (**C**) ROQ-C—*P. crustosum*; (**D**) OTA—*P. nordicum*; (**E**) ROQ-C—*P. roqueforti*; (**F**) OTA—*P. verrucosum*, at different temperature regimes (5–35 °C). Data were fitted by a Beta function (see Table 3 for details).

The best fitting of toxin production data as function of T was obtained by the Bete equation (Equation (1); [45]). Good results are reported for STC produced by *A. versicolor*, OTA produced by *P. nordicum* and *P. verrucosum*, PA produced by *P. crustosum* and ROQ-C produced by *P. crustosum* and *P. roqueforti* (Table 3, Figure 7), with *R*^2^ ≥ 0.94 for all fungi except *A. versicolor* (*R*^2^ = 0.727).

Regarding the other fungi/toxins, toxin production was possible only for a few temperature regimes (i.e., ROQ-C and MPA produced by *P. roqueforti*). Therefore, no data modeling was performed.

#### 2.5.2. Water Activity

The range of *a_w_* that allowed toxin production was limited to 0.93–0.99, with only 3 points available. Therefore, no modeling was applied to this dataset.

## 3. Discussion

The ripening of hard cheese is carried out commonly in a temperature range from 10 to 20 °C, as defined in the guidelines of many products Protected for Denomination of Origin, e.g., Fontina, Fiore Sardo and Emmentaler. For Italian grana type cheeses, the environmental conditions during aging are between 15 °C and 22 °C, as reported in the “Parmigiano Reggiano” and “Grana Padano” cheese production guidelines (www.politicheagricole.it). Grana cheeses are long—ripened high quality products; according to preliminary unpublished data, starting from 9 month storage, the *a_w_* varies between 0.92 and 0.85 in the crust and between 0.94 and 0.87 if a wider layer is considered, while the relative humidity of storehouses ranges between 72% and 88%.

Most of the fungi studied grew optimally around 25 °C, but for *P. citrinum* the relative growth was higher at 30 °C, and for *P. verrucosum* and *P. nordicum* at 20 °C. The susceptibility to *a_w_* regimes was considerable; *a_w_* = 0.99 was optimal for all species except the more xerophilic *A. versicolor*, which grew best with *a_w_* = 0.96. *A. versicolor*, *P citrinum*, *P. nalgiovense*, *P. roqueforti* and *P. verrucosum* grew down to 0.87 *a_w_*; *a_w_* = 0.90 was the limit for *P. nordicum* and *P. camemberti* and *a_w_* = 0.94 for *P. crustosum*, even at the longest incubation times considered in this study (56 days).

Therefore, all fungi studied can grow in the range of T common for the storage of hard, long maturing grana type cheeses. Recently Marin, et al. [47] reported *Penicillium* spp. as the dominant species on hard cheese; *Penicillium* and *Aspergillus* spp. were highly tolerant to water restriction, making them more competitive with other fungi during cheese ripening. This is confirmed in this study, where the lower a_w_ limit for growth was 0.87 for several *Penicillium* species and for *A. versicolor*.

Fungal growth in artificial media with modified *a_w_*, as managed in this study, possibly suffered from the high amount of salt supplemented in the lowest *a_w_* regimes. It is well know that compounds added to modify *a_w_* (glycerol or NaCl), interfere with fungal metabolism, the former enhancing fungal growth, being a carbon source, and the latter becoming toxic for fungi at high dosages [47,48,49]. Toxicity more than *a_w_* could have limited the fungal activity of some *Penicillium* species; therefore, further trials on cheese will be required to define cardinal *a_w_* regimes.

No toxin production is reported in literature for *P. nalgiovense*; this fungus was not considered for mycotoxin analysis, but only to check its potential competition with other fungi. It was noticed that it was more xerophilic than other *Penicillia* studied and showed more than 50% relative growth up to *a_w_* = 0.93 after 14 days of incubation. *Penicillium nalgiovense* and *P. crustosum* were the fastest growing fungi at 10 °C, *P. citrinum* and *P. nordicum* at 5 °C. Growth speed is important because it favors fungi competiveness. Therefore, these data suggest that *P. nalgiovense* could effectively compete with other fungi present in cheese, depending on the abiotic conditions of exposure.

As well-known from literature, the range of abiotic conditions that allows toxin production is commonly narrow than that allowing growth and this is confirmed in the present study. CPA and MPA production started at 10 °C, while *P. camemberti* and *P. roqueforti* grew from 5 °C. OTA production started at 10 and 15 °C, respectively for *P. nordicum* and *P. verrucosum*, while growth was observed from 5 °C. MPA was detected up to 20 °C, while growth continued up to 30 °C.

Water activity resulted as the most limiting factor for toxin production; in fact, growth was observed down to 0.87–0.90 *a_w_*, depending on the fungal species, while toxin production stopped at 0.93 *a_w_* for CIT, PR and ROQ-C, 0.99 *a_w_* for OTA by *P. verrucosum* and 0.96 *a_w_* for all the other fungi.

Even though toxic metabolites have been reported in *P. camemberti* and *P. roqueforti*, they are used as ripening agents, and CIT, CPA, MPA and ROQ-C were effectively detected in fungal cultures in the range of conditions considered in this study. Therefore, strains included in starter *inocula* should be preliminarily checked for toxigenicity. This can be inferred also from Dall’Asta, de Dea Lindner, Galaverna, Dossena, Neviani and Marchelli [44], who found OTA contamination increased duiring storage of blue cheeses.

CIT is the toxin detected in the highest amount in this study, around 3000 ng/mm^2^ of fungal colony, in *P. citrinum* grown at 35 °C and 0.99 *a_w_*. CIT was produced at all the temperature conditions considered, in agreement with Bailly, Querin, Le Bars-Bailly, Benard and Guerre [18], while it rapidly decreased from 1200 to 100 ng/mm^2^ when *a_w_* moved from 0.99 to 0.96 (T = 20 °C).

CIT production by *P. camemberti* is a matter of concern because it was detected at 5 °C, T typically applied for home storage by consumers. Fortunately, according to Manabe [50], few *P. camemberti* strains were able to produce CIT. No CIT was produced in the present study by *P. verrucosum*, even if previously reported [27,51]. This could be due to its susceptibility to salt concentration. According to Schmidt-Heydt, et al. [26], when salt concentration is equal to or above 20 g/L, *P. verrucosum* shifts from producing CIT to OTA. As the amount of NaCl used to modify the *a_w_* in our study was about 70 g/L minimum, lacking of CIT production at different *a_w_* regimes is not surprising.

PA, typically produced by *P. crustosum*, was detected in all the considered conditions except at 35 °C, with the optimum at 25 °C, in agreement with Larsen, et al. [52] and Kokkonen, et al. [53].

ROQ-C and MPA are typically *P. roqueforti* extrolites [23,24,27], but ROQ-C is also produced by *P. crustosum* [54,55]. ROQ-C was produced by *P. roqueforti* at all the considered conditions. Several authors agree on the very high incidence of toxigenic strains in *P. roqueforti* populations [23,24,56,57]. Significant reduction in ROQ-C was observed with T lower than 12 °C, NaCl concentrations 8% and modified atmosphere (1%–5% O_2_ and 20%–40% CO_2_; [48]). A substantial support in describing the role of abiotic factors will come from the genome of *P. roqueforti* recently published [58].

STC, a very stable compound [59], has so far only beendetected on the rind of hard cheeses [31]. In this study, STC was produced by *A. versicolor* over the T range 10–35 °C and with *a_w_* ≥ 0.96, but STC production with lower a_w_ cannot be excluded in cheese.

CPA was supposed to be produced by *P. citrinum* and by *P. camemberti*, but it was only detected in the latter colonies, incubated between 10 and 25 °C and with *a_w_* ≥ 0.96. The amount of CPA measured increased with temperature and *a_w_* increase, in agreement with Le Bars [19] who also confirmed the high incidence of toxigenic strains in *P. camemberti* populations.

*Penicillium nordicum* and *P. verrucosum* are OTA producers and in this study, as expected, *P. nordicum* was more efficient at slightly lower T and higher *a_w_*, 0.96–0.99, compared to *P. verrucosum*. The latter resulted toxigenic also at very low *a_w_*, 0.87–0.93, with a long incubation time, in agreement with Schmidt-Heydt et al. [60] and Schmidt-Heydt, et al. [26]. They also underlined the efficacy of salt addition in limiting OTA production by *P. nordicum*. Since the ambient T and the cheese rind *a_w_* during cheese ripening are favorable, possible contaminations by *P. nordicum* and *P. verrucosum* must not be underrated, even if OTA is sometimes undetectable [56].

Growth and toxin production rates by the studied fungi was well described by Bete function and by logistic regression in different T and *a_w_* regimes, respectively. The Bete equation is in agreement with good modelling results obtained by Rossi, et al. [61] for fungi involved in *Fusarium* head blight complex and deoxynivalenol and zearalenon production, by Nazari, et al. [62] for *Fusarium langsethiae*/*F. sporotrichioides* and T-2/HT-2 toxins production, by Battilani, et al. [63] to model *A. flavus* growth on maize and recently for *A. carbonarius* growth on grapes [64]. Other modeling approaches are described in literature to predict fungal growth as T function [65,66], but the use of Bete equation is more advisable when functions are developed to be used for mechanistic model development [67]. Furthermore, Bete equation was used to model other key steps of fungal infection cycle as function of T, like *A. flavus* sporulation [68] and *A. carbonarius* germination. However, further tests of growth on cheese will be necessary to develop a good model.

## 4. Conclusions

The highest risk of toxin production in cheese should occur between 15 and 25 °C, where 4 out of 8 of the species considered in this study had their optimal toxigenic activity. In particular, between 15 and 22 °C, 3 fungi, *P. crustosum*, *P. nordicum* and *P. roqueforti*, are expected to cause major problems, with OTA, PA, ROQ-C and MPA as expected toxins. STC and CIT should also be monitored, even if their production is optimized with higher T regimes. Regarding *a_w_*, if the cheese rind has an *a_w_* below 0.93, mycotoxin production should not be at very high risk, at least within the first 2 weeks of development of a spoiling mold. Nevertheless, some contamination data reported in literature after long ripening periods are in contrast with this statement. This study underlines two important factors regarding mycotoxin-producting fungi in cheese: (i) they can grow in conditions comparable with those used for cheese ripening and (ii) a multi-mycotoxin contamination of cheese is possible. Therefore, the models developed in this study should be validated/adjusted with data obtained on cheese. This will be a good starting point to develop a model to predict contamination by different mycotoxins. In the meantime, monitoring ripened cheese for toxin contamination is strongly suggested, especially when their presence will be predicted on the basis of abiotic and biotic data.

## 5. Materials and Methods

### 5.1. Fungal Strains

The fungi included in this study are all toxigenic, except for *P. nalgiovense*, which is however considered because of its prevalence in ripened pork meat and signaled in cheese, and as a possible competitor of toxigenic species. Characterized strains were purchased from CBS-NAW fungal collection (http://www.cbs.knaw.nl/; Table 5).

### 5.2. Culture Media

Ingredients for media were purchased from Himedia Laboratories (Mumbai, India).

Czapek Yeast Agar (CYA, [69]), supplemented with sodium chloride (NaCl) (Carlo Erba, Milan, Italy) to modify the original *a_w_* = 0.99, was used to perform the ecological trials (Table 6).

### 5.3. Inoculum Preparation, Inoculation and Incubation

A conidia suspension was prepared using 7-day old colonies grown on Malt Extract Agar, (MEA, [71]). The spores were collected using 20 mL of sterile bi-distilled water added to each Petri dish, gently agitating the culture to remove conidia. The suspension was adjusted to a concentration of 10^6^ spores/mL using an haemocytometer, in agreement with fungal CFU/g detected in naturally contaminated cheese. Then, 10 μL of conidial suspension of each strain was centrally inoculated in 60 mm Ø Petri plates and incubated in the proper conditions; the trial was managed in triplicate.

Two experiments were performed to assess the role of: (i) T and (ii) *a_w_* on fungal growth and mycotoxin production.

Regarding T, CYA inoculated plates, all prepared without any *a_w_* modification, were incubated at temperatures ranging between 0 °C and 40 °C, 5 ± 1 °C step, for 14 days.

Regarding *a_w_*, the CYA medium was adjusted to *a_w_* values from 0.87 to 0.99 step 0.03 *a_w_*, and incubated at 20 ± 1 °C for 14 days. Longer incubation times, up to 56 days, were considered for the *a_w_* regimes ≤0.90.

### 5.4. Fungal Growth Measurement

The inoculated Petri dishes were observed after 3, 7, 10 and 14 days, and two perpendicular colony diameters were measured; a weekly schedule was applied for longer incubation times (from 14 to 56 days). After 14 days of incubation (56 days for *a_w_* ≤ 0.90), the plates were sealed in plastic bags and stored at −20 °C before mycotoxin analysis.

### 5.5. Mycotoxin Analysis

Ochratoxin A, CPA, PR-toxin, MPA, ROQ-C, PA and CIT were considered as produced by the proper fungi (reported in Table 5); they were measured at the end of the incubation time (14 day old cultures).

#### 5.5.1. Reagents and Standards

The chemicals and solvents used for the extraction and clean-up solutions were ACS grade or equivalent (Carlo Erba, Milan, Italy). All the water used was de-ionized and, for HPLC, purified through a Milli-Q treatment system (Millipore, London, UK). For HPLC analysis, methanol and acetonitrile were HPLC grade (Merck, Darmstadt, Germany). Mycotoxin standards were obtained from Sigma-Aldrich (St. Louis, MO, USA) and Biopure (Tulln, Austria). Working standard solutions were prepared by dilution with acetonitrile and kept at −20 °C.

#### 5.5.2. Toxin Extraction

Toxin extraction was performed by putting the fungal colony and agar media in a flask containing 40 mL of acetonitrile. Then, the mix was vigorously shaken using a rotary-shaking stirrer for 1 h in order to smash the agar medium into little pieces, filtered (folded filter paper 595 ½, Whatman, Sigma-Aldrich, St. Louis, MO, USA) and diluted using the HPLC mobile phase before being analyzed.

#### 5.5.3. HPLC-MS/MS Analysis

The mycotoxins (STC, CPA, CIT, ROQ, MPA, OTA, PR toxin, PA), were analyzed using an HPLC-MS/MS system, consisting of a LC 1.4 Surveyor pump, a Quantum Discovery Max triple-quadrupole mass spectrometer (Thermo-Fisher Scientific, San Jose, CA, USA) and a PAL 1.3.1 sampling system (CTC Analitycs AG, Zwingen, Switzerland); the system was controlled by Xcalibur 1.4 software (Thermo-Fisher). The mycotoxins were separated on a Betasil RP-18 column (5 µm particle size, 150 × 2.1 mm, Thermo-Fisher); except for PA, a mobile-phase gradient water-acetonitrile (both acidified with 0.2% formic acid) from 65:35 to 25:75 in 6 min, then isocratic for 5 min was used; for PA, the mobile-phase gradient water-acetonitrile (both acidified with 0.2% formic acid) was from 40:60 to 10:90 in 5 min, then isocratic for 3 min. The flow rate was always 0.2 mL/min and the injection volume 20 µL. The ionization was carried out with an ESI interface (Thermo-Fisher) in positive mode as follows: spray capillary voltage 4200 kV, sheath and auxiliary gas 35 and 10 psi, respectively, temperature of the heated capillary 270 °C. The selected fragment ions and the parent ion [M]^+^ were: 310, 281 and 253 *m*/*z* for STC ([M]^+^ 325 *m*/*z*); 196, 182 and 140 *m*/*z* for CPA ([M]^+^ 337 *m*/*z*); 233, 205 and 191 *m*/*z* for CIT ([M]^+^ 251 *m*/*z*); 334, 322 and 193 *m*/*z* for ROQ-C ([M]^+^ 390 *m*/*z*); 303, 275 and 207 *m*/*z* for MPA ([M]^+^ 321 *m*/*z*); 358, 341 and 239 *m*/*z* for OTA ([M]^+^ 404 *m*/*z*); 279, 173, 161 and 145 *m*/*z* for PR toxin ([M]^+^ 321 *m*/*z*); 616, 558 and 332 *m*/*z* for PA ([M]^+^ 634). The collision energy was different for each mycotoxin (ranging from 15 to 33 V) and the argon collision pressure was 1.5 mTorr. Quantitative determination was performed using LC-Quan 2.0 software (Thermo-Fisher Scientific); LODs were 30 µg/L for MPA, CPA, PR-toxin, PA and STC, 20 µg/L for ROQ-C, 10 µg/L for CIT and OTA.

### 5.6. Data Analyses

Statistical analyses were performed using SPSS v.23 (SPSS Inc., Armonk, NY, USA, 2012). Mycotoxin production data were statistically compared by using a OneWay-ANOVA Test transforming all values by y = ln before analysis to homogenize the variance. Tukey test was applied to highlight significant differences between means.

Data on fungal growth, intended as the fungal culture diameter, at different T or *a_w_* regimes, were considered separately for each incubation time. They were standardized (rated on the maximum value observed), to obtain relative growth in a 0–1 scale, with 0 = no growth, and 1 = maximum growth. Relative growth of each fungus, at all incubation times, were jointly analyzed. Thirty six mean values were used for T (9 T regimes and 4 incubation times, mean of 3 replicates) and 20 values for *a_w_* (5 *a_w_* regimes and 4 incubation times, mean of 3 replicates). The same approach was applied to obtain relative mycotoxin production. Different nonlinear regression models were fitted to the rate data in order to describe fungal growth and mycotoxin production as function of T and *a_w_*; the equation parameters were estimated applying the non-linear regression procedure of the statistical package PASW SPSS statistics v.23 (SPSS Inc., Armonk, NY, USA, 2012) which minimizes the residual sum squares using the Levenberg-Marquardt algorithm. The best model was chosen based on the adjusted R^2^ and on the number of iterations required by the algorithm to converge on parameter estimates, as indicators of goodness of fit.

Minitab 17 (Minitab Inc., State College, PA, USA) was used to develop the surface response contour plots of data, in relation to the combinations T × time of incubation and *a_w_* × time of incubation, for each considered fungus. For each combination T × time or *a_w_* × time, relative growth, computed as previously described, was used as input for data plotting (relative growth values were transformed from 0–1 scale to 0–100 scale to satisfy the type of data input requested by Minitab). Two-dimension surface response contour plots were drawn, with five quoted lines (contour levels): 0%, 25%, 50%, 75% and 100% of the relative growth. This kind of data presentation is considered useful when many data are collected in the study and it is not easy to report all data and to compare and comment results.

In order to represent the combined effect of T and *a_w_*, the equations developed to describe fungal growth as function of these two variables were combined and the 50% relative growth values obtained for each fungus were plotted.

## Figures and Tables

**Table 1:** Spoiling agents reported in cheese, mycotoxin produced and their toxic effect.

Fungi	Mycotoxin	Toxic Effect	Reported By
***Aspergillus* spp.**	Sterigmatocystin (STC)	Carcinogenic, mutagenic [28]	[29,30,31,32]
***P. brevicompactum***	Mycophenolic acid (MPA)	Mutagenic, possible acute toxicity [33]	[34]
***P. camemberti***	Cyclopiazonic acid	Neurotoxic, possible acute toxicity [28]	[19,35]
***P. citrinum***	Citrinin (CIT)	Nephrotoxic, teratogenic [36,37]	[18,38]
***P. crustosum***	Penitrem A (PA)	Neurotoxic [28]	[21]
***P. expansum***	Patulin	Carcinogenic, mutagenic, teratogenic, harmful to liver, possible acute toxicity [28]	[39,40]
***P. roqueforti*, *P. crustosum***	Roquefortine C (ROQ-C)	Neurotoxic [28]	[40,41]
***P. roqueforti***	PR Toxin	Mutagenic, Carcinogenic [42]	[39,43]
***P. verrucosum*, *P. nordicum***	Ochratoxin A (OTA)	Nephrotoxic, carcinogenic, hepatotoxic [28]	[44]

**Table 2:** Fungal maximum colony diameter (cm) reached after 10 days of incubation at the reported temperature or water activity (*a_w_*), in Experiment 1 and 2, respectively.

Species	Experiment 1. Temperature *		Experiment 2. Water Activity **	
	*Diameter Max (cm)*	*Temperature (°C)*	*Diameter Max (cm)*	*a_w_*
***A. versicolor***	3.1	25	3.1	0.96
***P. camemberti***	4.3	25	4.0	0.99
***P. citrinum***	4.2	30	3.1	0.99
***P. crustosum***	5.1	25	5.0	0.99
***P. nalgiovense***	4.8	25	4.1	0.99
***P. nordicum***	3.7	20	3.3	0.99
***P. roqueforti***	5.5	25	5.5	0.99
***P. verrucosum***	4.0	20	4.0	0.99

* In experiment 1.Temperature ranged between 0 °C and 40 °C, 5 ± 1 °C step and media *a_w_* = 0.99; ** In experiment 2. Water activity ranged between 0.87 and 0.99, step 0.03 *a_w_* and T = 20 ± 1 °C

**Table 3:** Parameters of the equations developed to calculate relative growth and mycotoxin production for the selected fungi. The Bete and the logistic equations were used to describe fungal growth respectively as function of temperature (T) and water activity (*a_w_*). The Bete equation was also used to describe relative mycotoxin production as function of T.

Fungi	Variable	T_min_/T_max_	Estimated Parameters (*Standard Error*)			
			*a*	*b*	*c*	*R^2^*
**Relative Growth**						
***A. versicolor***	T	5/35	6.85 (*0.353*) *	2.09 (*0.122*)	0.63 (*0.122*)	0.964
	*a_w_*		−32.719 (*3.478*)	−36.351 (*3.958*)	0.953 (*0.039*)	0.956
***P. camemberti***	T	5/40	3.22 (*0.150*)	0.72 (*0.049*)	4.10 (*0.779*)	0.823
	*a_w_*		29.12 (*156.829*)	−24.07 (*11.615*)	198.77 (*32,649.12*)	0.925
***P. citinum***	T	5/40	8.03 (*0.387*)	2.33 (*0.114*)	1.12 (*0.132*)	0.961
	*a_w_*		55.72 (*8.565*)	−59.74 (*9.294*)	1.04 (*0.053*)	0.966
***P. crustosum***	T	5/40	3.95 (*0.379*)	1.01 (*0.115*)	1.24 (*0.274*)	0.798
	*a_w_*		32.69 (*8.161*)	−34.57 (*9.079*)	1.22 (*0.233*)	0.927
***P. nalgiovense***	T	5/40	4.70 (*0.245*)	1.25 (*0.074*)	1.56 (*0.209*)	0.899
	*a_w_*		59.29 (*9.254*)	−64.24 (*10.1*)	1.01 (*0.044*)	0.970
***P. nordicum***	T	0/35	5.15 (*0.200*)	1.50 (*0.063*)	1.41 (*0.14*)	0.900
	*a_w_*		32.72 (*3.478*)	−36.35 (*3.958*)	0.94 (*0.039*)	0.956
***P. roqueforti***	T	5/40	4.26 (*0.262*)	1.08 (*0.083*)	2.45 (*0.498*)	0.902
	*a_w_*		73.08 (*44.606*)	−75.11 (*47.168*)	1.28 (*0.601*)	0.967
***P. verrucosum***	T	5/35	5.01 (*0.573*)	1.80 (*0.250*)	0.44 (*0.043*)	0.936
	*a_w_*		58.61 (*8.441*)	−63.15 (*9.176*)	1.03 (*0.043*)	0.979
**Relative Mycotoxin Production**						
***A. versicolor* STC**	T	5/35	6.31 (*1.842*)	1.95 (*0.659*)	0.57 (*0.386*)	0.727
***P. crustosum* PA**	T	5/35	4.78 (*0.457*)	1.31 (*0.145*)	2.19 (*0.607*)	0.942
***P. crustosum* ROQ-C**	T	5/35	5.14 (*0.330*)	1.43 (*0.103*)	1.53 (*0.258*)	0.981
***P. nordicum* OTA**	T	5/35	3.65 (*0.138*)	0.87 (*0.042*)	1.79 (*0.185*)	0.991
***P. roqueforti* ROQ-C**	T	5/35	5.19 (*0.440*)	1.48 (*0.139*)	0.79 (*0.161*)	0.986
***P. verrucosum* OTA**	T	5/35	6.30 (*0.224*)	1.72 (*0.066*)	0.87 (*0.074*)	0.998

* Standard error of parameters were reported in parenthesis.

**Table 4:** Mycotoxin production under different regimes of temperature (T) and water activity (*a_w_*) after 14 days of incubation. Data are reported as mean ng of toxin produced per mm^2^ of fungal colony area (ng/mm^2^; three replicates).

		*A. versicolor*		*P. camemberti*			*P. citrinum*		*P. crustosum*				*P. nordicum*		*P. roqueforti*					*P. verrucosum*		
T (°C)	*a_w_*	STC		CIT	CPA		CIT		PA		ROQ		OTA		MPA		ROQ		PR	CIT	OTA	
*Temperature*																						
**5**	0.99	<LOD ^#^	*a* ^†^	1.1	<LOD	*a*	0.9	*a*	7.4	*b*	6.7	*a*	<LOD	*a*	<LOD	*a*	<LOD	*a*	<LOD	<LOD	<LOD	*a*
**10**	0.99	7.4	*bc*	1.0	0.5	*a*	66.8	*b*	31.7	*c*	237.4	*b*	0.2	*a*	0.9	*c*	112.3	*b*	<LOD	<LOD	<LOD	*a*
**15**	0.99	16.9	*c*	0.7	0.4	*a*	63.8	*b*	150.9	*e*	630.8	*bc*	4.0	*b*	1.7	*d*	246.9	*c*	<LOD	<LOD	0.8	*b*
20 *	0.99	81	*d*	3.8	7.6	*b*	1187.8	*c*	152.7	*e*	679.5	*c*	9.0	*b*	0.4	*b*	324.9	*d*	<LOD	<LOD	4.6	*d*
**25**	0.99	91.4	*d*	0.5	20.3	*c*	1147.5	*c*	197.2	*e*	824.3	*c*	3.7	*b*	<LOD	*a*	282.0	*d*	<LOD	<LOD	12.6	*e*
**30**	0.99	284	*e*	<LOD	<LOD	*a*	1728.9	*c*	81.8	*d*	615.6	*bc*	0.1	*a*	<LOD	*a*	248.0	*c*	<LOD	<LOD	2.5	*c*
**35**	0.99	6.6	*b*	<LOD	<LOD	*a*	2918.1	*c*	<LOD	*a*	<LOD	*a*	<LOD	*a*	<LOD	*a*	0.1	*a*	<LOD	<LOD	<LOD	*a*
*Water activity*																						
20	**0.93**	<LOD		<LOD	<LOD		733		25. 6		149.0		<LOD		<LOD		68.2		<LOD	<LOD	<LOD	
20	**0.96**	4.4		1.0	0.3		100.2		59.6		644.9		0.8		21.1		335.5		<LOD	<LOD	<LOD	

* data collected at 20 °C and 0.99 *a_w_* are common for the trial at different T and different *a_w_* regimes. ^#^ LOD is specific for each mycotoxin. See materials and methods for details. ^†^ Different letters define significant difference according to Tukey test (*p* ≤ 0.01).

**Table 5:** Fungal strains and related mycotoxins considered in this study.

Species	Code	Mycotoxin (Abbreviation)
*A. versicolor*	CBS 108959	Sterigmatocystin (STC)
*P. camemberti*	CBS 122399	Citrinin (CIT), Cyclopyazonic Acid (CPA)
*P. citrinum*	CBS 122396	CIT, CPA
*P. crustosum*	CBS 115503	Penitrem A (PA), Roquefortine C (ROQ-C)
*P. nalgiovense*	CBS 109609	#
*P. nordicum*	CBS 112573	Ochratoxin A (OTA)
*P. roqueforti*	CBS 221.30	Mycophenolic Acid (MPA), ROQ-C, PR Toxin (PR-TOXIN)
*P. verrucosum*	CBS 325.92	CIT, OTA

# No toxins are reported for this fungus.

**Table 6:** Amounts of sodium chloride (NaCl) added to Ckzapek Yeast Agar (CYA) to modify medium water activity (*a_w_*; [70]).

NaCl (g/100 mL)	*a_w_*
**7.01**	0.96
**11.98**	0.93
**16.56**	0.90
**19.40**	0.87
**23.55**	0.84
**30.10**	0.81
**39.90**	0.78
